# Light people: Professor Mengkun Liu

**DOI:** 10.1038/s41377-026-02211-x

**Published:** 2026-03-16

**Authors:** Wenjun Zheng, Siqiu Guo

**Affiliations:** 1 Department of Physics and Astronomy, Stony Brook University, Stony Brook, NY 11794 USA; 2 Light Publishing Group, Changchun Institute of Optics, Fine Mechanics and Physics, Chinese Academy of Sciences, Changchun, 130033 China; 3 Ji Hua Laboratory, Foshan, 528200 China

## Abstract

At the frontier where light meets quantum matter, Professor Mengkun Liu has built a distinctive research program that integrates advanced optical nanoscopy, quantum materials, and emergent light–matter interactions. As a Professor in the Department of Physics and Astronomy at Stony Brook University, he leads the Ultrafast & Near-field Infrared Laboratory (UNI-Lab), where his group pioneers infrared-to-terahertz near-field spectroscopy under extreme conditions, enabling direct visualization of polaritons, collective excitations, and quantum phenomena at the nanoscale. Over the past decade, Professor Liu and his collaborators have not only made fundamental discoveries in quantum materials and nanophotonics but have also reshaped experimental capabilities through instrument innovation - ranging from magneto-near-field optical microscopy to synchrotron-based infrared nanoscopy. In this interview, he reflects on the scientific ideas that drive his research, the philosophy behind building a creative research group, and his perspective on the future of optical science at the quantum frontier.

**Figure:**
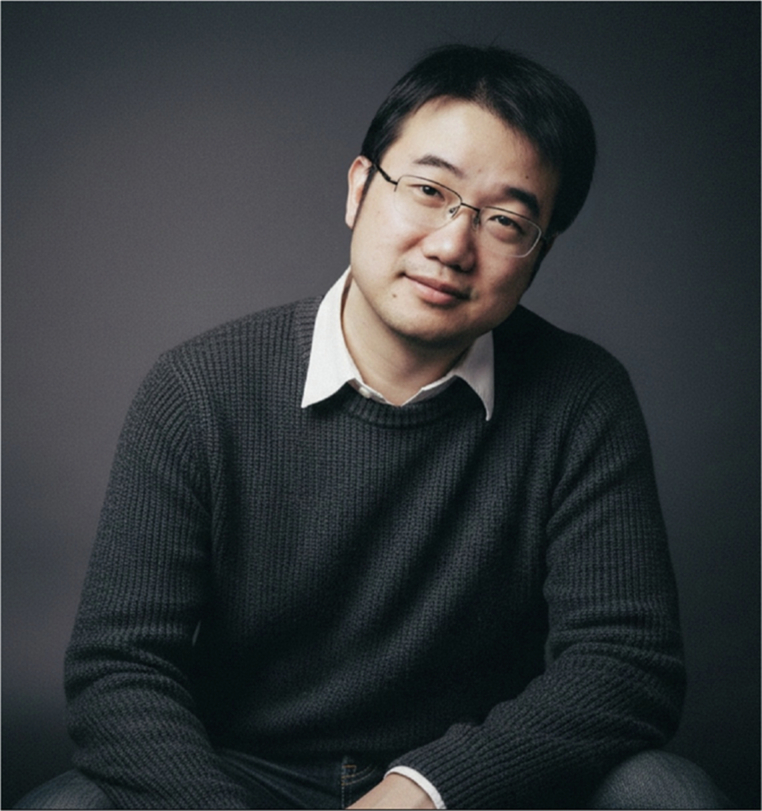
Prof. Mengkun Liu

**Short Bio:** Mengkun Liu is a Professor of Physics and Astronomy at Stony Brook University, USA. His research focuses on experimental condensed matter physics, quantum materials, and advanced optical spectroscopy and nanoscopy. He received his Ph.D. in physics at Boston University and subsequently worked as a postdoc researcher at UC San Diego. He also holds a joint appointment at Brookhaven National Laboratory, where he played a pivotal role in developing synchrotron-based infrared instrumentation. His group is widely recognized for innovation and research in the field of ultrafast optics, near-field techniques, and quantum materials research. Professor Liu is also actively involved in mentoring young scientists and in developing next-generation experimental platforms for quantum science.

**Q1. Could you briefly introduce your current research directions and the central questions your group is trying to answer?**

My group is interested in how collective electronic, lattice, and photonic degrees of freedom emerge, interact, and evolve in quantum materials when they are confined to the nanoscale. Many of the most intriguing phenomena in condensed matter such as polaritons, superconductivity, magnetism, and topological states are inherently collective and often highly inhomogeneous. Yet, for a long time, we have relied on spatially and temporally averaged probes to study them.

This limitation is precisely what motivates our approach. We build unique optical platforms via combining infrared-to-terahertz light with scanning probe nanoscopy, ultrafast laser techniques, and extreme conditions such as low temperature and high magnetic field. This allows us to directly visualize how quantum excitations emerge, propagate, interfere, and dissipate energy in real space and time. Currently we are particularly interested in developing quantum sensing nanoscopy platforms based on Quantum Hall effect and superconductor photon detectors.

**Q2. Near-field nanoscopy has become a defining element of your work. What makes this approach so powerful compared with conventional optical techniques?**

The key advantage of near-field nanoscopy is that it breaks the diffraction limit while retaining low-energy (meV to eV range) spectroscopic information^1–4^. This combination is essential for quantum materials, where relevant length scales—such as domain sizes, coherence lengths, and edge states—are often tens of nanometers or smaller. See Fig. 1 as a conceptual schematic of the technique under developement.

**Fig. 1:**
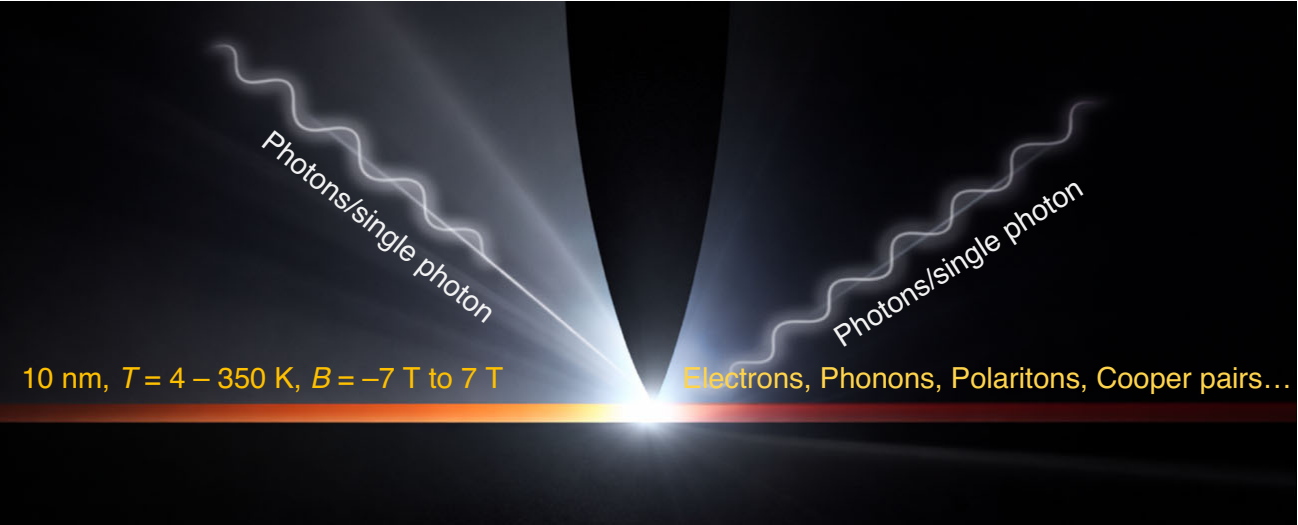
My group develops extreme-condition infrared-to-terahertz nanoscopy as a quantitative platform for probing collective and electronic excitations in quantum materials. We focus on regimes where conventional optics fails: deep subwavelength confinement, cryogenic temperature, high magnetic field, and ultralow signal levels. By integrating synchrotron-based nano-IR, THz near-field microscopy, and bolometric superconducting detection, we aim to directly access a variety of quantum phenomena including quantum Hall and superconducting excitations beyond far-field observables. Our long-term goal is to establish nanoscale spectroscopy as a primary tool for quantum materials and quantum device characterization under extreme conditions

Equally important is that near-field techniques are inherently local probes. They do not just tell us what excitations exist, but where they live and how they interact with their environment. In recent years, with our collaborators at Columbia University (Prof. D. N. Basov) and Brookhaven National Laboratory [Dr. G. L (Larry) Carr, Dr. Lukas Wehmeier], we have pushed this further by integrating magnetic fields, cryogenic operation, and broadband spectroscopy into the system, which has allowed us to explore regimes that were previously inaccessible. In this sense, near-field nanoscopy is evolving from a qualitative imaging tool into a quantitative research platform for condensed matter research. In addition, we are developing AI-enhanced analysis tools that can quantify the local dielectric function of materials with less than 3-5% error in general^5,6^.

**Q3. Your work often bridges optics, condensed matter physics, and materials science. How do you view interdisciplinary research?**

I tend to think that disciplines are conveniences rather than boundaries imposed by nature. Light does not know whether it is probing a “physics” or a “materials science” problem. What matters is whether we are aiming at the right problems and whether we have the appropriate tools to answer them. Many of our ideas originate from conversations across fields: sometimes with theorists, sometimes with materials growers, and sometimes with engineers. I greatly appreciate all the discussions with my collaborators and friends, although often the time it may not lead to any “concrete output” such as a research paper. Interdisciplinary research, in my mind, is about building a deep core expertise while remaining intellectually open. That openness often leads to unexpected connections and, occasionally, entirely new research directions. For example, we have used the near-field-enhancement of light to do nanoscale “photolithography” on silk-based materials^7^. More recently, our work on Bolometric Superconducting Optical Nanoscopy (BOSON) has helped bridge superconducting quantum sensing and polariton research, opening potential pathways toward quantum-correlated polaritonic circuits in the near future^8^.

**Q4. Instrumentation development has played a major role in your career. Why is building new tools so important to you?**

Many transformative advances in physics have followed the invention of new experimental tools. In my view, instrumentation is not secondary to discovery. It is often the enabler of discovery. When we develop a new microscope or spectroscopy platform, we are not simply improving resolution or sensitivity; we are fundamentally expanding the space of questions that can be meaningfully asked.

For instance, the advent of angle-resolved photoemission spectroscopy (ARPES) made it possible to directly map electronic band structures and many-body renormalizations, which proved essential for understanding unconventional superconductivity, correlated electron systems, and topological phases. Similarly, low-temperature scanning tunneling microscopy (STM) enabled real-space access to superconducting gaps, quasiparticle interference, and atomic-scale electronic inhomogeneity, revealing physics that had been completely hidden in momentum- or ensemble-averaged probes.

Similarly, the ability to perform low-energy optical nanoscopy under magnetic fields and at cryogenic temperatures opens new experimental regime. It allows us to directly visualize Landau quantization, collective mode propagation, symmetry-broken phase transitions, and edge or domain-localized optical or photothermal excitations with nanometer resolution. These phenomena were previously inferred mostly from bulk measurements or transport signatures. This level of access is particularly important for quantum materials, where edges or nonlocal interactions often play a decisive role at low photon energy scale in the order of several meVs.

I therefore see instrumentation as a long-term investment. In a fast-paced academic environment, this may appear risky, but to me it is a necessary commitment. It can take years to design, build, and mature a new experimental platform, yet once established, it often enables an entire ecosystem of scientific exploration—frequently extending well beyond the questions originally envisioned. Many of the most consequential breakthroughs emerge precisely from this patience: from taking the time to sharpen the tools before using them to probe new physics^9–11^.

**Q5. Your recent work increasingly involves terahertz frequencies and quantum materials. What excites you most about this regime?**

The terahertz regime occupies a uniquely important position in the energy landscape of quantum materials. With photon energies in the meV range, terahertz radiation directly addresses the low-energy collective excitations that ultimately determine macroscopic quantum behavior—plasmons, phonons, magnons, superconducting amplitude and phase modes, and other emergent quasiparticles.

What makes this regime particularly exciting is that many central concepts in modern condensed matter physics—topology, quantum geometry, and symmetry breaking—manifest themselves most clearly at these low energy scales. For example, topological gaps and flat bands, Berry-curvature–induced responses, and interaction-driven mass generation often appear in the meV range, where terahertz spectroscopy provides direct, nonperturbative access. In this sense, terahertz probes are not merely complementary to optical or transport measurements; they are often the most natural way to interrogate the relevant physics.

Terahertz techniques are also exceptionally powerful for studying magneto-related phenomena. Under applied magnetic fields, characteristic energy scales such as cyclotron resonance, Landau-level transitions, magnetoplasmons, and magnon/spin-wave excitations typically fall squarely in the terahertz range. This makes terahertz radiation ideally matched to resolving magnetic-field-induced gaps, Landau level quantization, and collective mode hybridization—particularly in low-dimensional and strongly correlated systems at cryogenic temperatures.

What excites me most is that terahertz nanoscopy allows us to access these excitations not only spectrally, but also spatially. By combining meV energy resolution with nanometer-scale imaging, we can directly visualize how quantum geometry, topological responses, and magneto-excitations evolve in real space—along edges, across domains, or near defects—where many of the most interesting phenomena actually reside. This capability is crucial for understanding dissipation, coherence, and nonequilibrium dynamics in quantum materials^11–13^. See Fig. 2 as a photo of the synchrotron THz-IR nanoscope as a user facility developed at BNL NSLS-II.

**Fig. 2:**
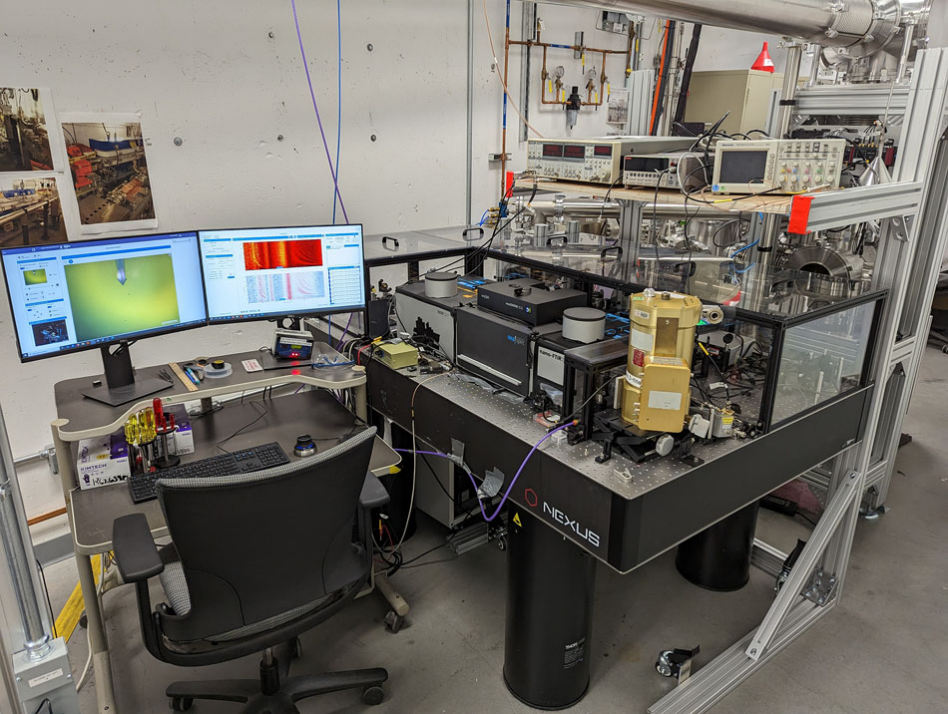
A multi-THz nanoscope at NSLS-II at Brookhaven National Laboratory as a user facility. A cryo-version is currently also under development

**Q6. As a group leader, how do you foster creativity and independence among your students and postdocs? What advice would you give to young researchers entering the field of optics and condensed matter physics?**

I try to create an environment where people feel comfortable exploring ideas and, just as importantly, making mistakes. In our group, asking “simple,” “naïve,” or even “stupid” questions is very much encouraged. Very often, those questions reveal hidden assumptions and can lead to the most interesting discussions. I also see learning as a collective process; we are constantly learning from each other and our collaborators, myself included.

At the same time, I place a strong emphasis on independence. Young researchers need to develop their own scientific taste, intuition, and judgment. They have to develop their own unique skill set that cannot be easily replaced by others (now including AI!). My role is not to micromanage, but to provide guidance, context, and a general sense of direction, while giving them enough freedom to take ownership of their projects. In fact, my proudest moments are not simply when my students publish a paper, but when they challenge my assumptions and prove me wrong—because that is when I know they are ready to lead their own paths as scientists, whether in academia or industry. Ultimately, I believe the success of a research group should not be measured only by publications, but by whether its members grow into confident, thoughtful, and capable scientists—and human beings.

For young researchers entering optics and condensed matter physics, my advice is to stay curious and be patient. Always be patient. Meaningful progress in science often takes longer than expected, and setbacks are part of the process. At the same time, try not to lose your sense of wonder or your sense of humor. Many of the most exciting problems initially look confusing, messy, or even contradictory. When mistakes are made, please just laugh them off. In the end, whether one becomes a scientist or chooses a different career path is less important. What matters most, in my view, is to continually cultivate curiosity and find a path that genuinely fulfills it. At the time of AI and robotics, this might be proven to become more and more important.

**Q7. You have published extensively and serve the community in multiple ways. What, in your view, makes a strong scientific paper?**

This is a difficult but important question. Researchers inevitably encounter papers published in high-profile journals that, at least in hindsight, feel less substantial than their visibility might suggest. This has always been the case and will likely remain so in the near future. It does not particularly bother me, because science is a human endeavor and no system is perfect.

What matters to me is the test of time. When I consider publishing a paper, I ask myself whether it will still be relevant in twenty years. That question matters more to me than where the paper appears. A strong paper, in my view, must begin with a clear and meaningful question. Technical sophistication, advanced instrumentation, or large data sets are not sufficient on their own. I have learned this the hard way more than once. There must be a genuine insight, a conceptual advance, or a new way of seeing a problem. This is an ideal I constantly aim for, although I do not pretend that I always succeed. Many projects fall somewhere between ambition and reality, and recognizing that gap is part of being honest with oneself as a scientist.

I also believe strongly in the data itself. If a result is truly important, it should largely speak for itself. Excessive framing, over-polished narratives, or fashionable wording can sometimes obscure rather than clarify the underlying physics. Clear communication is, of course, essential, but there is a fine line between clarity and hype. In my view, the best papers are those that present data faithfully, in the simplest and most logical way possible, and then explain, honestly and clearly, what the authors believe the data means in physical terms. That is really all it needs to do.

In the long run, papers that influence a field are often not those that maximize short-term impact, but those that introduce new ways of thinking, new experimental capabilities, or new questions that others can build upon. That said, this is a very high standard, and not an easy one to meet. For this reason, I do encourage my own students to publish in good journals, but always with the understanding that the “game” is far beyond publishing itself.

**Q8. Looking ahead, what do you see as major opportunities or challenges in optical science and nanophotonics?**

In today’s quantum and AI world, optical probes will inevitably play a central role not only in characterization, but also in control, readout, and data processing. Light provides a uniquely versatile interface, capable of addressing quantum states across a wide range of energy, length, and time scales. In this context and in terms of major opportunities, I often resonate with a remark by Professor Savas Dimopoulos at Stanford University:

*“The thing that differentiates scientists is purely an artistic ability to discern what is a good idea, what is a beautiful idea, what is worth spending time on, and most importantly, what is a problem that is sufficiently interesting, yet sufficiently difficult, that it hasn’t yet been solved, but the time for solving it has come now.”*

I think this perspective is particularly relevant today, as we are not lacking in technology or challenge, but we need to choose the right problems to pursue.

Looking ahead, I am especially excited about opportunities at the intersection of nanophotonics and superconducting quantum technologies. Platforms such as our Bolometric Superconducting Optical Nanoscopy (BOSON) approach aim to use ultrasensitive superconducting detectors to probe extremely weak optical and terahertz signals, opening new possibilities for studying superconducting qubits, on-chip quantum circuits, and their coupling to photons at the nanoscale^8^ (See also Fig. 3). These capabilities may enable direct insight into dissipation, decoherence, and energy flow in quantum devices, which remain central challenges for scalable quantum technologies.

**Fig. 3:**
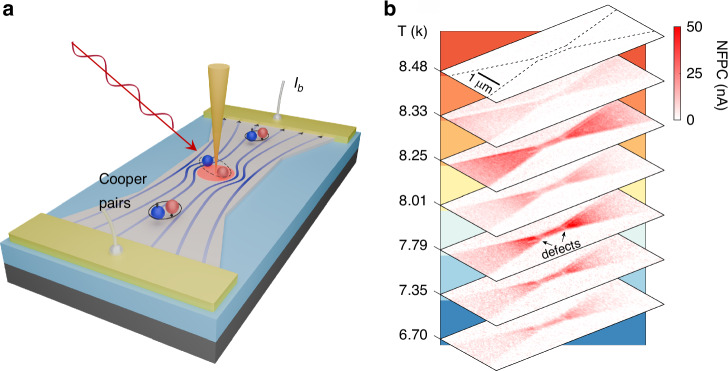
Near-field photocurrent imaging of superconducting transport using BOSON. **a** Schematic of near-field photocurrent nanoscopy, where localized optical excitation perturbs Cooper-pair transport and current redistribution in a superconducting device. **b** Temperature-dependent near-field photocurrent maps across the superconducting transition in a Nb device, revealing the emergence and evolution of spatially inhomogeneous local superconductor transition edges at the nanoscale

More broadly, optical nanoscopy offers a promising route toward exploring single-photon devices and quantum entanglement in real space. Being able to locally probe and manipulate light–matter interactions at the nanoscale may allow us to study how entangled states form, propagate, and degrade in realistic, imperfect environments. In this sense, nanophotonics is moving beyond simple imaging and spectroscopy, toward becoming an active tool for engineering and understanding quantum systems^14^.

Overall, I see this as an exciting period for the field. Looking back at how rapidly experimental capabilities have evolved, it is clear to me that nano-optics has moved beyond being a passive probe and is now deeply embedded in quantum science, influencing how we study nature and how we imagine future technologies.
